# Helix Matrix Transformation Combined With Convolutional Neural Network Algorithm for Matrix-Assisted Laser Desorption Ionization-Time of Flight Mass Spectrometry-Based Bacterial Identification

**DOI:** 10.3389/fmicb.2020.565434

**Published:** 2020-11-12

**Authors:** Jin Ling, Gaomin Li, Hong Shao, Hong Wang, Hongrui Yin, Hu Zhou, Yufei Song, Gang Chen

**Affiliations:** ^1^NMPA Key Laboratory for Quality Control of Therapeutic Monoclonal Antibodies, Shanghai Institute for Food and Drug Control, Shanghai, China; ^2^Department of Biochemical Drugs and Biological Products, Shanghai Institute for Food and Drug Control, Shanghai, China; ^3^Department of Analytical Chemistry, Shanghai Institute of Materia Medica, Chinese Academy of Sciences, Shanghai, China; ^4^Department of Gastroenterology, Lihuili Hospital of Ningbo Medical Center, Ningbo, China

**Keywords:** matrix-assisted laser desorption ionization-time of flight mass spectrometry, bacterial identification, helix matrix transformation, convolutional neural network, algorithm study

## Abstract

Matrix-assisted laser desorption ionization-time of flight mass spectrometry (MALDI-TOF MS) analysis is a rapid and reliable method for bacterial identification. Classification algorithms, as a critical part of the MALDI-TOF MS analysis approach, have been developed using both traditional algorithms and machine learning algorithms. In this study, a method that combined helix matrix transformation with a convolutional neural network (CNN) algorithm was presented for bacterial identification. A total of 14 bacterial species including 58 strains were selected to create an in-house MALDI-TOF MS spectrum dataset. The 1D array-type MALDI-TOF MS spectrum data were transformed through a helix matrix transformation into matrix-type data, which was fitted during the CNN training. Through the parameter optimization, the threshold for binarization was set as 16 and the final size of a matrix-type data was set as 25 × 25 to obtain a clean dataset with a small size. A CNN model with three convolutional layers was well trained using the dataset to predict bacterial species. The filter sizes for the three convolutional layers were 4, 8, and 16. The kernel size was three and the activation function was the rectified linear unit (ReLU). A back propagation neural network (BPNN) model was created without helix matrix transformation and a convolution layer to demonstrate whether the helix matrix transformation combined with CNN algorithm works better. The areas under the receiver operating characteristic (ROC) curve of the CNN and BPNN models were 0.98 and 0.87, respectively. The accuracies of the CNN and BPNN models were 97.78 ± 0.08 and 86.50 ± 0.01, respectively, with a significant statistical difference (*p* < 0.001). The results suggested that helix matrix transformation combined with the CNN algorithm enabled the feature extraction of the bacterial MALDI-TOF MS spectrum, which might be a proposed solution to identify bacterial species.

## Introduction

Matrix-assisted laser desorption/ionization time-of-flight mass spectrometry (MALDI-TOF MS) is a fast, inexpensive and reliable tool for the identification of bacteria, and it has become a gold standard for microbial identification in clinical microbiology laboratories within the last decades (Lasch et al., 2009; Bryson et al., 2019; Hou et al., 2019; Welker et al., 2019). As a spectrum-recognition-based method, the classification algorithm plays a critical role in the process (Fangous et al., 2014). The similarity evaluation system for the MALDI-TOF MS spectra of bacteria is commonly used in routine analysis. Standard spectra are acquired from multiple measurements of a single defined strain so that the biological variability of strains is captured and the impact from the random sampling of MALDI-TOF MS is removed. Sample spectra are compared with the standard spectrum library by calculating the similarity among multiple parameters, such as peak positions, intensities and frequencies, thus ensuring the highest possible levels of accuracy and reproducibility across a complete range of microorganisms (Wang et al., 2018; Rotcheewaphan et al., 2019). Then, a matching score is obtained. The results of potential species with a matching score above a set threshold will be listed and sorted by the scores. The Biotyper software (Bruker Daltonik GmbH, Bermen, Germany), a typical example of a similarity evaluation system, is widely used in both routine analysis and scientific research. The standard spectrum library can be extended by users to identify more species of bacteria. However, only a small number of attributes in MALDI-TOF MS spectra such as the peak height and peak area are analyzed and empirically linked to microbial species in a similarity evaluation system (Weis et al., 2020). Therefore, some challenging species with similar MS peaks, such as *Shigella* and *E. coli* species are difficult to be identified by traditional algorithm (Ling et al., 2019).

To fully exploit the MALDI-TOF MS spectrum features, machine learning algorithms have been used to refine species identification (Mather et al., 2016; Kim et al., 2019). Many types of machine learning algorithms, such as the support vector machine (SVM) and random forest (RF), have been applied to optimize bacterial identification. De Bruyne and colleagues used the SVM and RF to binarize the MALDI-TOF MS spectra of the genera *Leuconostoc*, *Fructobacillus*, and *Lactococcus*, and the method achieved excellent discriminatory performance (De Bruyne et al., 2011). The SVM algorithm was also used to discriminate methicillin-resistant (MRSA) from methicillin-sensitive *S. aureus* (MSSA) based on their MALDI-TOF MS spectra. An artificial neural network, a high performance machine learning algorithm, was employed to conduct the rapid and accurate identification of *Bacillus fragilis* and some of its subgroups (Zhang et al., 2004; Lasch et al., 2009). In the previous study, a short-term culture method was presented to induce over expression of new proteins as biomarkers which can be detected using MALDI-TOF MS (Ling et al., 2019). The dimensionalities of the full spectra were reduced using a isomap non-linear dimensionality reduction algorithm to fit the BPNN’s input requirement. After that, a neural network algorithm was employed as a classifier for MS spectrum identification. The back propagation neural network (BPNN) model achieved great success in distinguishing *Escherichia coli* and *Shigella* species. The prediction accuracy of the BPNN model was 97.71% with the novel culture approach. However, the multi-class classification of species using the BPNN model was not achieved because there was no spectral feature extraction process.

Recently, convolutional neural networks (CNNs) have achieved great success in image classification, object recognition and natural language processing (Hsieh et al., 2020). Unlike other machine learning algorithms, the convolutional layers in CNNs extract image feature information from source images to form a weight map during the training process, which provide more feature details than manual acquisition (Wang et al., 2020). Fully connected layers are an essential component of CNNs, which have been proven to be very successful in image classification. The features broken down from images are fed into a fully connected neural network structure that drives the final classification decision. Seemingly, the MALDI-TOF MS spectrum is an image. In fact, the data form of the MALDI-TOF MS spectrum is an one-dimensional (1D) array of intensity values, which is drawn as a line chart. An 1D array data type is a structure that contains an ordered collection of data elements in which each element can be referenced by its ordinal position in the collection. The data elements and their ordinal positions serve as critical attributes of 1D array-type data, which are equivalent to peak intensity and peak location in original MALDI-TOF MS spectrum.

In this study, we present a novel helix matrix transformation combined with CNN algorithm for the multi-class classification of species. Helix matrix is a kind of inerratic matrix in mathematics. The helix matrix transformation was suggested in order to convert 1D array-type MALDI-TOF MS spectrum data into image-like matrix-type data for CNN model training for the first time. The spectrum was converted into an image (matrix-type data) with some black and gray blocks after the helix matrix transformation. The correlation between peaks in original spectrum was established when folding 1D array-type data in two dimensions. The smaller parts of the image, black and gray block groups in each view, were new spectrum features, which were characteristic of MS peak and peak correlation in original MALDI-TOF MS spectrum. Then, the CNN algorithm was employed, which successfully classified 14 bacterial species based on their MALDI-TOF MS spectra. The convolution layer “scanned” the image with a convolution kernel to extract features which may be important for classification. Afterward, the features were downsampled, and then the same convolutional structure repeated again. The convolution identified successively features and sub-features from the original image and its sub-parts. Eventually, the process of convolution identified the essential features which can help to classify the image.

## Materials and Methods

### Bacterial Strains

A total of 14 bacterial species (58 strains) including *E. coli* (20 strains), *Staphylococcus aureus* (20 strains), *Staphylococcus capitis* (1 strain), *Staphylococcus sciuri* (1 strain), *Staphylococcus vitulinus* (1 strain), *Staphylococcus xylosus* (1 strain), *Staphylococcus epidermidis* (2 strains), *Staphylococcus simulans* (1 strain), *Staphylococcus haemolyticus* (1 strain), *Staphylococcus hominis* (1 strain), *Salmonella* (5 strains), *Kocuria rhizophila* (1 strain), *Staphylococcus lentus* (1 strain), and *Enterococcus faecalis* (2 strains) were selected for the experiment. The information of the experimental strains is shown in Supplementary Table 1. Ten species in all belonged to *staphylococcus* with close relationships, which increased the difficulty of classification.

### Culture Condition and Sample Preparation

The strains were incubated on commercial tryptic soy agar (Huankai microbial, Guangzhou, China) at 35°C for 24 h to obtain fresh colonies. The fresh colony was extracted with 60 μL of 70% formic acid (Sigma-Aldrich, Louis, United States) and 60 μL of acetonitrile (Merck, Darmstadt, Germany) with a vortex for 30 s. After the centrifugation of the extracting solution at 10000 g for 3 min, 1 μL of the supernatant was loaded onto a MALDI target plate spot and left to dry. Each sample spot was overlaid with 1 μL α-Cyano-4-hydroxycinnamic acid (CHCA) (5 mg/mL) (Sigma-Aldrich, Louis, United States) in a 50:48:2 acetonitrile:water:trifluoroacetic acid (Tedia, Fairfield, United States) matrix solution and was dried at room temperature.

### MALDI-TOF MS Analysis

The MS analyses were performed using a 4800 Plus MALDI-TOF/TOF^TM^ (Applied Biosystems, Framingham, MA, United States). The mass spectrometer was externally calibrated before use. The mass error parameter of calibration was set as 50 ppm. Each MS spectrum was obtained by summing 50 acceptable sub-spectrums obtained in random sampling mode with a fixed laser intensity of 3500 for the MS analysis. The raw data were collected from 2000 to 12,000 *m/z* in the linear positive-ionization mode. The peak detection parameters were set as follows: Signal/Noise >20, local noise window width = 250 *m/z* and minimum peak width at full width half max = 2.9 *m/z*.

### Dataset Preparation

Each MALDI-TOF MS spectrum was preprocessed with noise removal and baseline correction using the Data Explore software (Ab Sciex, Redwood City, United States), followed by it being exported into an individual text file. The text file contained the numeric value of the intensity for every single point of the MS spectrum. To manage the bulk data, these numeric values of the intensity in text files were read and normalized to a range from 0 to 255 using Python v3.7.4, then compacted into 2,500 points and inserted into a MySQL v5.7.20 (MySQL AB, Sweden) data table with some basic information, such as species, strain, and date of analysis. Numeric labels of data from 0 to 13 were assigned to each species. Before modeling, all MS numeric value data were exported with labels in line into a text file to obtain high loading performance.

### Data Transformation

Here, we present a helix matrix transformation for the array of an MS spectrum, which makes 1D array-type spectrum data into matrix-type. Firstly, a square helix matrix was created using the formula as follows:

(1) $$M=\left\{ \begin{array}{c} u_{n,k-n+1}^{\left(n\right)}=u_{n-1,k-n+1}^{\left(n-1\right)}+1\\ u_{i,k-n+1}^{\left(n\right)}=u_{i-1,k-n+1}^{\left(n-1\right)}+1,\\ i=n+1,n+2,\ldots ,k-n\\ r_{k-n+1,k-n+1}^{\left(n\right)}=r_{k-n,k-n}^{\left(n\right)}+1\\ r_{k-n+1,j}^{\left(n\right)}=r_{k-n+1,j+1}^{\left(n\right)}+1,\\ j=k-n,k-n-1,\ldots ,n+1\\ d_{k-n+1,n}^{\left(n\right)}=d_{k-n+1,n+1}^{\left(n\right)}+1\\ d_{i,n}^{\left(n\right)}=d_{i+1,n}^{\left(n\right)}+1,\\ i=k-n,k-n-1,\ldots ,n+1\\ r_{n,n}^{\left(n\right)}=r_{n+1,n}^{\left(n\right)}+1\\ r_{n,j}^{\left(n\right)}=r_{n,j-1}^{\left(n\right)}+1,\\ j=n+1,n+2,\ldots ,k-n \end{array}\right.,i=2,\ldots ,k/2$$

where *k* is the number of elements on the matrix side, *n* is the ordinal of the square from outside-in of the helix matrix, and *i* and *j* are row number and column number, respectively.

If *k* was an odd integer, the center of the helix matrix was set using the equation as follows:

(2) $$M_{\left(k+1\right)/2,\left(k+1\right)/2}=M_{\left(k+1\right)/2,\left(k-1\right)/2}+1$$

The numeric values of 2,500 points of the MS spectrum were clockwise rolled into the square helix matrix with a 50 × 50 size using the equation as follows:

(3) $$M_{i,j}=A_{M_{i,j}}$$

where *A* is the data array of the 2,500 points of the MS spectrum.

To remove the low intensity noise and peaks, image binarization was carried out using the formula as follows:

(4) $$\text{dst}\left(x,y\right)=\left\{ \begin{array}{cc} \text{maxValue} & \text{if}\text{src}\left(x,y\right)>T\\ 0 & \text{otherwise} \end{array}\right.$$

where *T* is the threshold value.

A bicubic interpolation over a 4 × 4 pixel neighborhood resize method was selected to resize the images. The equations were as follows:

(5) W(x)={(a+2)|x|3-(a+3)|x|2+1for|x|≤1a|x|3-5a|x|3+8a|x|-4afor 1<|x|<20otherwise

(6) $$f\left(x,y\right)=f\left(x_{i},y_{j}\right)W\left(x-x_{i}\right)W\left(y-y_{j}\right)$$

where *a* is a factor, and *i* and *j* are image channels. In our study, the parameters are set as follows: *a* = −0.5, *i* = 0, and *j* = 0.

The data visualization after each step was performed using the Matplotlib library. The data labels were converted into one-hot labels using the Keras library. The dataset containing all numeric values and labels was split randomly into a training dataset and validation dataset with a split ratio of 0.8, which means that 80% of the data was used for model training and the other 20% was used for model validation. The test dataset was created using 1000 additional MS spectra of each species followed by helix data transformation. These spectra were never used before to be a test set.

### Convolutional Neural Network Modeling

All training and evaluations were carried out on a Dell T7820 workstation equipped with two Intel Xeon Gold 5118 CPUs, 64 Gb of DDR4 RAM and two Nvidia GTX1080Ti graphics cards. CUDA v10.0, a parallel computing platform and programming model developed by NVIDIA for general computing on graphical processing units (GPUs) was installed for two GTX1080Ti graphics cards. The operating system was the 64-bit CentOS Linux system v7.5. The CNN models were constructed using TensorFlow v2.0.0, which is widely used for building and training artificial neural network models. The NVIDIA CUDA Deep Neural Network library (cuDNN) v7.4.2, a GPU-accelerated library of primitives for deep neural networks was used for creating model. The cuDNN provides highly tuned implementations for standard routines such as forward and backward convolution, pooling, normalization, and activation layers.

As shown in Supplementary Table 2, the CNN model contains 3 convolutional layers, 2 batch normalization layers, 1 max pooling layer, 1 fully connected layer, and 1 Softmax layer to form the output prediction. The numbers of filters were set as 4, 8, and 16 for the three convolutional layers, respectively. The kernel size was set as 3. The numbers of nodes in the fully connected layer and output layer were 128 and 14, respectively. The activation function of the convolutional layer and output layer were the rectified linear unit (ReLU) function and Softmax function, respectively. The Softmax function defined in Eq. (7) was applied in the last layer to produce the prediction probability over the 14 output classes (Hsieh et al., 2020).

(7) $$f{\left(s\right)}_{i}=\frac{e^{s_{i}}}{\sum_{j=1}^{C}e^{s_{j}}}$$

where *s*_*i*_ are the scores inferred by the net for each class in *C*.

The categorical cross-entropy was selected as the loss function, which was defined in Eq. (8). The goal of the network is to minimize CE.

(8) $$\text{CE}=-log\frac{e^{s_{p}}}{\sum_{j}^{C}e^{s_{j}}}$$

where *s*_*p*_ is the CNN score for the positive class.

Adam is selected as the optimizer. The hyper-parameters ß1 and ß2 are 0.9 and 0.999, respectively. The learning rate is set as 0.001 and the number of epochs was set as 1.

### Back Propagation Neural Network Modeling

To investigate the benefits of data transformation and convolutional layers in our algorithm, a back propagation neural network (BPNN) was created by removing the data transformation step and convolutional layers (see Supplementary Figure 1). The BPNN models were trained and evaluated using the same environment and library as that of CNN. The input size was set as 2,500 to fit the data array of the original spectrum. The numbers of nodes in the fully connected layer and output layer, the loss function, the optimizer, the learning rate and the number of epochs were set the same as those of the CNN model.

### Model Evaluation

The loss, precision, accuracy and recall were selected to evaluate the model training since they are commonly used in most cases for evaluations. The loss values were calculated using categorical cross-entropy formula mentioned above. The precision, accuracy and recall were calculated as follows:

(9) $$\text{Precision}=\frac{tp}{tp+fp}$$

(10) $$\text{Accuracy}=\frac{tp+tn}{tp+tn+fp+fn}$$

(11) $$\text{Recall}=\frac{tp}{tp+fn}$$

where *tp* is true positives, *fp* is false positives, *tn* is true negatives, and *fn* is false negatives.

A confusion matrix was established to investigate the classification performance. Each row of the matrix stands for a predicted label while each column represents a true label. The receiver operating characteristic (ROC) curve was drawn with the true positive rate and false positive rate.

## Results

**FIGURE 1:**
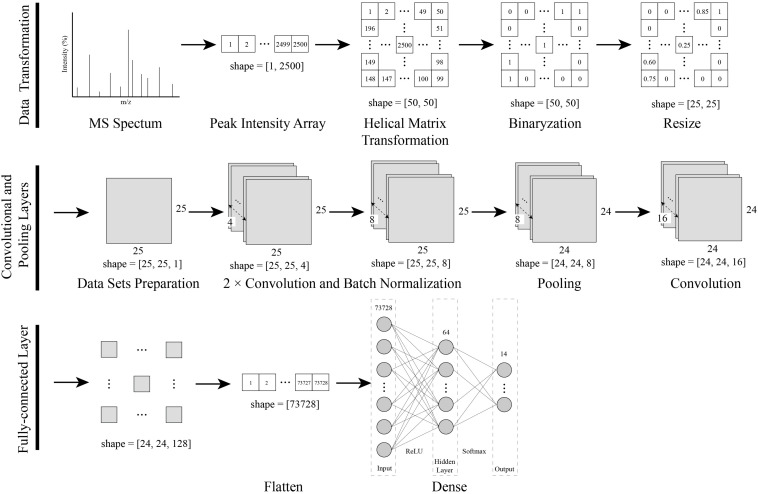
Procedures of the data transformation combined with the CNN modeling. The one-dimensional MS spectra were converted into a two-dimensional matrix with a novel helix matrix transformation method. The two-dimensional matrix data were binarized and resized, and then compressed into a dataset for CNN training. Finally, a CNN model with convolutional, pooling and, dense layers was created and trained with a dataset.

As shown in Figure 1, a data transformation and CNN modeling approach was established for the identification of bacteria using MALDI-TOF MS.

### Data Transformation

**FIGURE 2:**
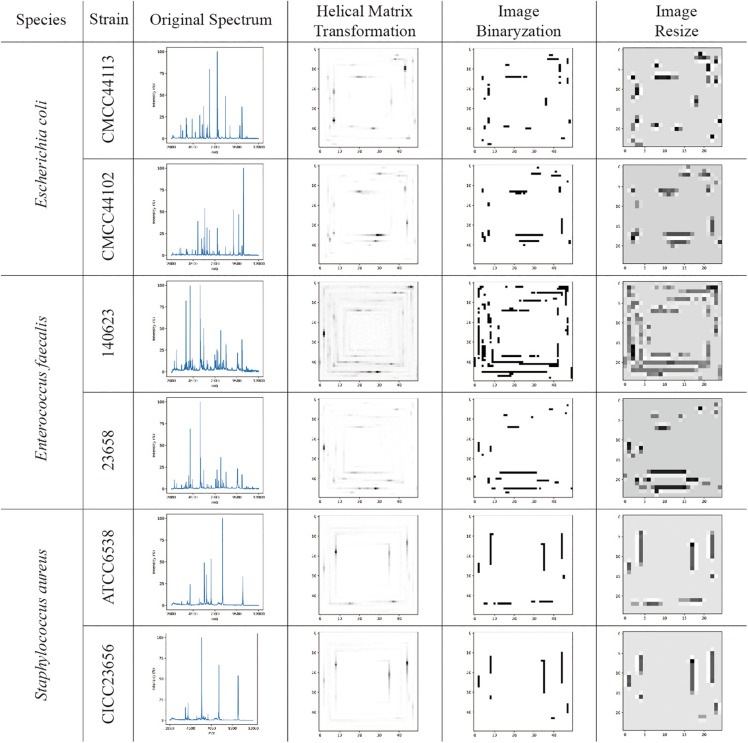
The visualization examples of original MS spectra; images converted by the helix matrix transformation; images after binarization; and resized images of *Escherichia coli*, *Enterococcus faecalis*, and *Staphylococcus aureus* species, respectively. The lines with various shades of gray to black shown in the images are the MS peaks after data transformation.

The visualizations of the helix matrix transformation are shown in Figure 2, which provided insights into how transformation works and how the features of the MS spectrum were revamped after transformation. As shown in Figure 2, the original MS spectrum was 1D array-type data. After the helix matrix transformation, the MS data of the strains were rolled similar to a Swiss roll into a matrix-type data with a size of 50 × 50. The MS peaks were transformed into lines with various shades of gray to black depending on their intensity, which kept the profile of the spectrum. To remove the low intensity noises and peaks, binarization was performed using threshold segmentation. The *T* threshold was set as a maximum value which makes all peaks in spectra detected by DataExplore software involved. Firstly, peak list I was obtained using DataExplore software, and peak list II was obtained from helix matrix transformed image after filtering with *T* threshold value. The *T* threshold value was decided by comparing the peak list II with the peak list I. After the parameter optimization, the threshold for binarization was set as 16 and the final size of the matrix-type data was set as 25 × 25 to obtain a clean dataset with a small size in order to greatly reduce the computational burden (data not shown). The bicubic interpolation method was used to prevent adjacent lines from being joined together. The features in the 2D image (matrix-type data) were obviously preserved after resizing, as shown in Figure 2.

### Model Evaluation

**FIGURE 3:**
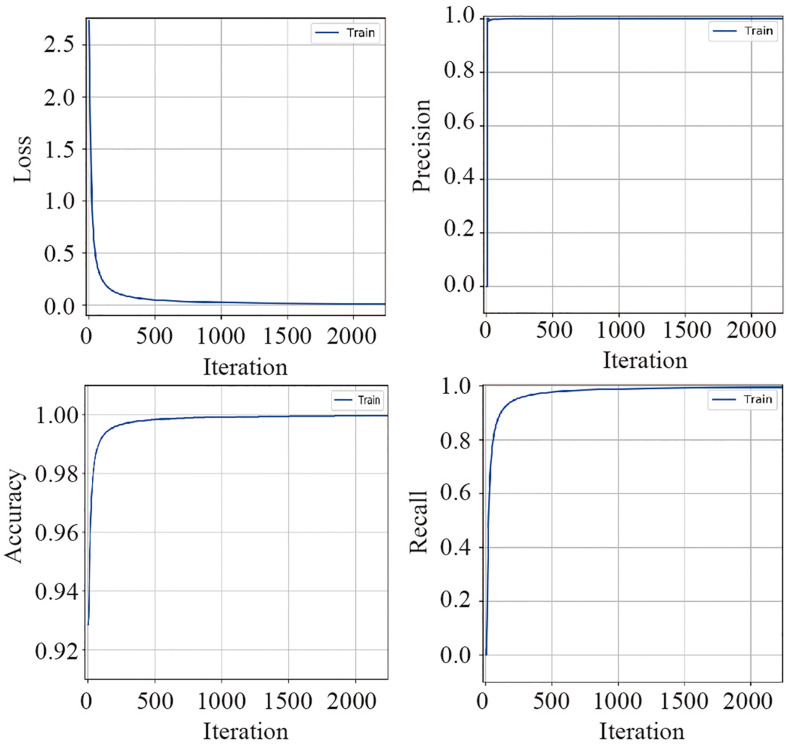
The valuation parameter curves of the CNN model’s training using the validation dataset. The loss, precision, accuracy, and recall curves are plotted according to their values in each iteration.

**FIGURE 4:**
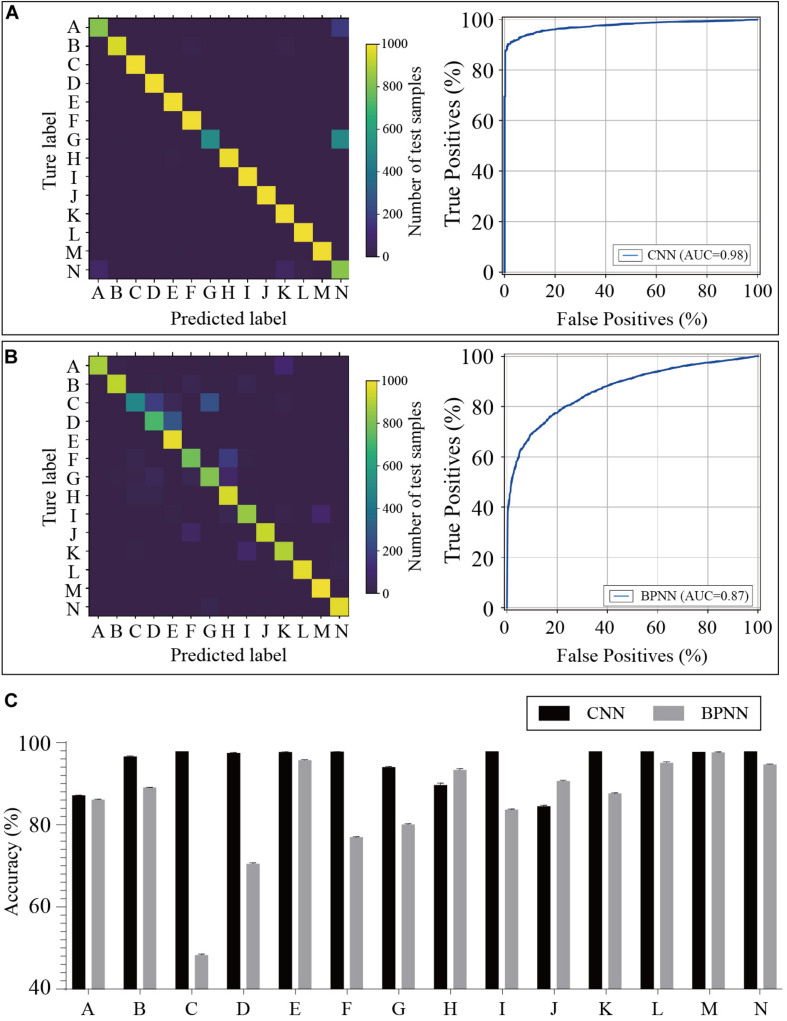
Predicted results of the bacterial species based on the CNN and BPNN models. Confusion matrices and receiver operating characteristic curves of the CNN **(A)** and BPNN **(B)** models are plotted based on the extent of matching between the predicted labels and true labels. **(C)** The accuracies are calculated by the prediction model using test samples. CNN, convolutional neural network. BPNN, back propagation neural network. The annotation of labels A to N related to the species that are listed in Supplementary Table 1.

The training dataset including 67,200 MS spectra was used for model training while the validation dataset including 16,800 MS spectra was used for model validation. A total of 2,400 iterations were carried out in 1 epoch. The loss curve of the training is shown in Figure 3. The loss values were 2.9561, 0.0418, 0.0269, and 0.0187 at the points at the beginning, after 500 iterations, after 1000 iterations, and after 1500 iterations, respectively. The loss value holds steady after 1500 iterations. At the end of the training, the loss value, accuracy, precision, and recall were 0.0126, 0.9996, 0.9977, and 0.9962, respectively, which indicated the model was well trained (Figure 3). The test set including a total of 14,000 MS spectra (1000 MS spectra for each species) with labels was used to test the prediction performance of the CNN and BPNN models. Figure 4A shows the confusion matrix and ROC curve of the prediction results based on the CNN model. In the confusion matrix, the diagonal shows the percent of correctly predicted records for each species and the off-diagonals show the percentage of misclassifications for each species. The classification accuracy for the 12 species was close to 100%, which suggested high classification performance for the CNN model. The overview of the ROC curves is shown in Figure 4A. The area under curve (AUC) value was 0.98. The confusion matrix and ROC curve of the predicted results based on the BPNN model are shown in Figure 4B. The AUC value of ROC curve was 0.87. The predicted accuracies of the CNN and BPNN models for each species are shown in Figure 4C. The accuracies of the CNN and BPNN models were 97.78 ± 0.08 and 86.50 ± 0.01, respectively, with a difference (*p* < 0.001) that supports a difference between the two accuracy results. These results suggested that the helix matrix transformation combined with the CNN model algorithm achieves better classification performance in bacterial identification based on MALDI-TOF MS.

## Discussion

Matrix-assisted laser desorption ionization-time of flight mass spectrometry is a rapid, high-throughput identification method for bacterial identification, which has been successfully applied in clinical microbiology laboratories (Schubert and Kostrzewa, 2017; Cordovana et al., 2018). The classification algorithm for classifying a bacterial MS database plays a critical role in the identification approach (Fangous et al., 2014; Mesureur et al., 2018). Manufacturer-provided software, such as FlexAnalysis and ClinProTools from Bruker Daltonics, are widely used for classification (Epperson et al., 2018; Rahi and Vaishampayan, 2019). A large proportion of classification studies were performed using FlexAnalysis and ClinProTools with preprogrammed machine learning algorithms including the SVM, spiking neural network (SNN), and quantum clustering (QC) (Weis et al., 2020; Delavy et al., 2019). The preprogrammed algorithms are easy to use, but they restrict the development of new algorithms.

Recently, CNNs have achieved great success in image classification in daily use and have also been applied in scientific studies (Hochuli et al., 2018; Hsieh et al., 2020; Zhou et al., 2018). A novel helix matrix transformation method was suggested to convert 1D array-type MS spectrum data into matrix-type. Because the peaks were standing in a row in the original spectrum, a very close distance between two adjacent peaks would reduce the recognition of the spectrum, which may cause low bacterial identification accuracy. In addition, the MS peaks are considered as independent protein types in some traditional algorithms. The correlation between peaks is ignored. After the helix matrix transformation, the distances of the peaks in the part of the low *m/z* range at the periphery of the matrix were extended, which increased the recognition in the spectrum. Meanwhile, the helix transformation also gave the correlation in space between two peaks in low and high *m/z* ranges. These changes balanced the spatial distribution of peaks, which revamped the profile of the MS spectrum. The binarization process removed the low intensity noises and peaks so that the classifier would focus on the major features of data. The threshold value of binarization can be set lower to obtained more information for distinguishing species with similar spectra.

The proposed CNN structure extracts the low-level features of an image with 2D convolutional filters in earlier layers and more complex features in deeper layers, which allowed the model to learn complex image differences (Zhou et al., 2018). Meanwhile, the BPNN can only use fully connected layers for classification. Therefore, the CNN is better than BPNN in multi-class spectrum classification.

In algorithm studies, public data sets are commonly used to test whether the algorithm can work on the type of object. For examples, MNIST and CIFAR datasets are well-known for deep learning research (training and testing neural network model) (Ferré et al., 2018). The MNIST is a dataset of handwritten digits. It has 60,000 training samples and 10,000 test samples. CIFAR-10 is an established computer-vision dataset used for object recognition. The CIFAR-10 dataset consists of 60000 of 32 × 32 color images in 10 classes, with 6,000 images per class. Since there is no public data set of MALDI-TOF MS spectrum on bacteria for deep learning research, we created an in-house dataset refers to the number of categories and data volume of MNIST and CIFAR-10 (shown in Supplementary Table 1). Then, the CNN and BPNN models were created and evaluated using the in-house dataset with 14 classes of bacterial species. Ten of the fourteen species with close relationship belong to staphylococcus, which increase the difficulty of classification. When conducting classification using the BPNN model, the AUC value of the ROC curve was 0.87. The value was significantly increased to 0.98 using the helix matrix transformation combined with the CNN algorithm. The predicted accuracies of the CNN and BPNN models for each species had a statistical difference (*p* < 0.001) according to a *t*-test. These results suggested that the helix data transformation combined with the CNN algorithm has a better classification ability and can solve the multi-classification problems for MALDI-TOF MS-based identification of bacteria.

In summary, we presented a novel method that combined a helix data transformation with a CNN algorithm for MALDI-TOF MS-based identification of bacteria. The code can be downloaded at https://github.com/ttelva/HMTCNN.git.The helix matrix transformation converted the 1D array-type MS spectrum of bacteria into matrix-type data with the original spectrum profile. An in-house dataset with 84,000 of MALDI-TOF MS spectra was built for training the neural network model. The algorithm was proved to be successfully applied in bacterial identification using an independent test dataset with 14,000 MS spectra. We also compared our algorithm with BPNN and the results indicate that helix matrix transformation combined with convolution provide a better performance of classification. In the following research, more species will be selected to train a model for the routine identification of bacteria in a laboratory.

## Data Availability Statement

The raw data supporting the conclusions of this article will be made available by the authors, without undue reservation.

## Ethics Statement

The studies involving human participants were reviewed and approved by the Ethics Committee of Ningbo Medical Center Lihuili Hospital. The patients/participants provided their written informed consent to participate in this study.

## Author Contributions

GC, HS, YS, and JL contributed to the conception and design of the study. JL, GL, HW, and HY performed the MALDI-TOF MS analysis. JL organized the database and wrote the draft of the manuscript. GC and HZ contributed to the manuscript’s revision. All authors contributed to the article and approved the submitted version.

## Conflict of Interest

The authors declare that the research was conducted in the absence of any commercial or financial relationships that could be construed as a potential conflict of interest.

## Supplementary Material

Supplementary Figure 1 Schematic view of the BPNN model structure.
