# Supplementary figures and images for: Helix Matrix Transformation Combined With Convolutional Neural Network Algorithm for Matrix-Assisted Laser Desorption Ionization-Time of Flight Mass Spectrometry-Based Bacterial Identification
Source: Front Microbiol. 2020 Nov 12;11:565434. doi: 10.3389/fmicb.2020.565434 (PMC7693542; doi:10.3389/fmicb.2020.565434)

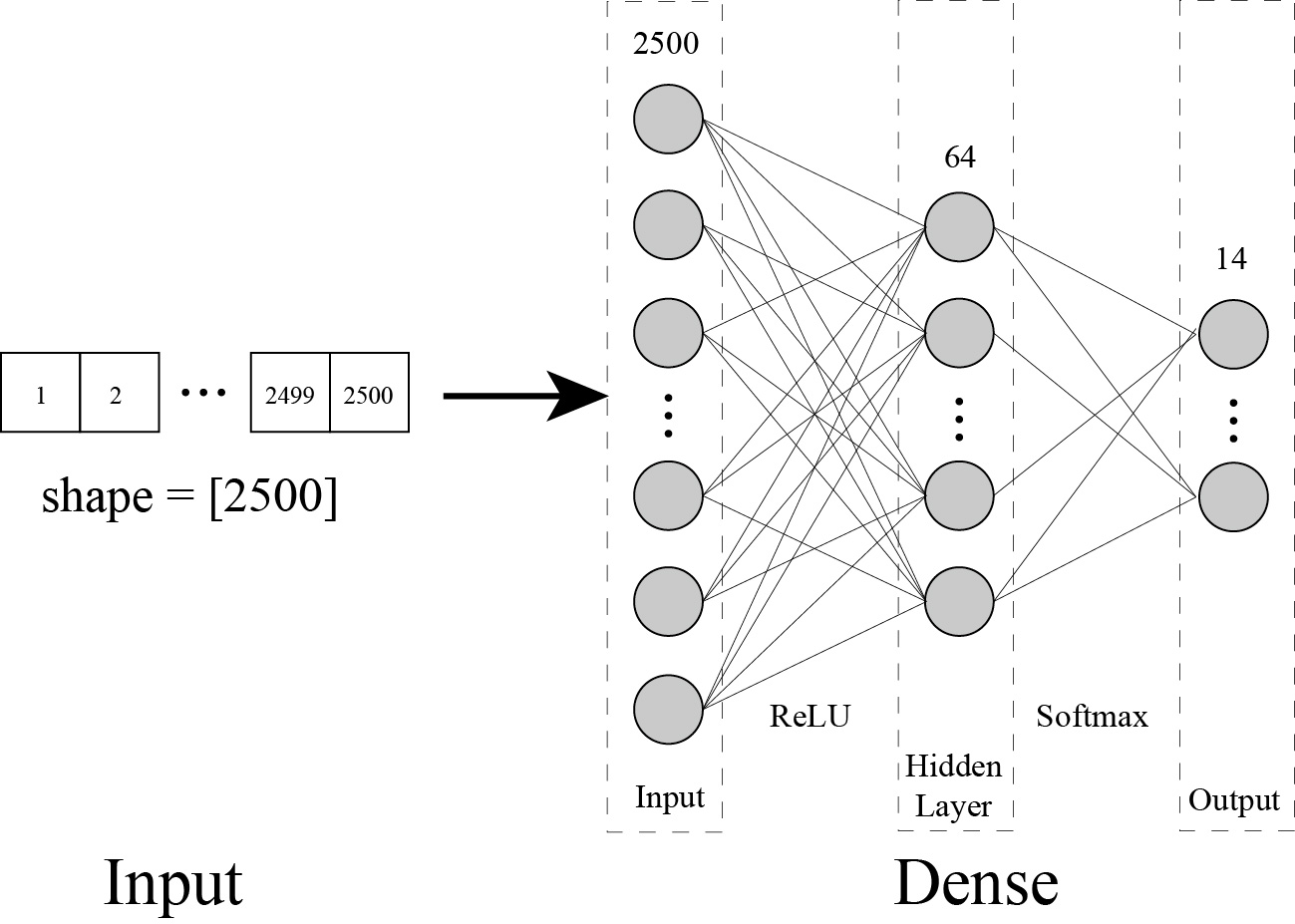

Supplement: Supplementary Figure 1 — Schematic view of the BPNN model structure. [file Image_1.JPEG]
