# Supplementary material for: Helix Matrix Transformation Combined With Convolutional Neural Network Algorithm for Matrix-Assisted Laser Desorption Ionization-Time of Flight Mass Spectrometry-Based Bacterial Identification
Source: Front Microbiol. 2020 Nov 12;11:565434. doi: 10.3389/fmicb.2020.565434 (PMC7693542; doi:10.3389/fmicb.2020.565434)
Supplement: Supplementary file 2 [file Table_1.docx]

**Table S1.** Basic information of the experimental strains

| Species | Strains | Source | Label | Number of spectra for training and validation | Number of spectra for prediction | |
| --- | --- | --- | --- | --- | --- | --- |
| *Enterococcus faecalis* | 23658 | Reference strain | A | 3000  3000 | | 500 |
|  | 140623 | Reference strain |  |  |  | 500 |
| *Staphylococcus aureus* | LHL40230 | Isolated strain | B | 300 | | 50 |
|  | LHL38966 | Isolated strain |  | 300 | | 50 |
|  | LHL39092 | Isolated strain |  | 300 | | 50 |
|  | LHL39727 | Isolated strain |  | 300 | | 50 |
|  | LHL38648 | Isolated strain |  | 300 | | 50 |
|  | LHL38766 | Isolated strain |  | 300 | | 50 |
|  | LHL39689 | Isolated strain |  | 300 | | 50 |
|  | LHL38133 | Isolated strain |  | 300 | | 50 |
|  | LHL39536 | Isolated strain |  | 300 | | 50 |
|  | LHL39312 | Isolated strain |  | 300 | | 50 |
|  | LHL39289 | Isolated strain |  | 300 | | 50 |
|  | LHL39210 | Isolated strain |  | 300 | | 50 |
|  | LHL37386 | Isolated strain |  | 300 | | 50 |
|  | LHL37022 | Isolated strain |  | 300 | | 50 |
|  | LHL37415 | Isolated strain |  | 300 | | 50 |
|  | LHL38825 | Isolated strain |  | 300 | | 50 |
|  | CICC23656 | Reference strain |  | 300 | | 50 |
|  | ATCC6538 | Reference strain |  | 300 | | 50 |
|  | CMCC26003 | Reference strain |  | 300 | | 50 |
|  | YS4 | Isolated strain |  | 300 | | 50 |
| *Staphylococcus capitis* | CICC21723 | Reference strain | C | 6000 | | 1000 |
| *Staphylococcus sciuri* | CICC23471 | Reference strain | D | 6000 | | 1000 |
| *Staphylococcus vitulinus* | CICC10850 | Reference strain | E | 6000 | | 1000 |
| *Staphylococcus xylosus* | CICC22112 | Reference strain | F | 6000 | | 1000 |
| *Staphylococcus epidermidis* | CMCC26069 | Reference strain | G | 3000 | | 500 |
|  | YS5 | Isolated strain |  | 3000 | | 500 |
| *Staphylococcus simulans* | YS1 | Isolated strain | H | 6000 | | 1000 |
| *Staphylococcus haemolyticus* | YS2 | Isolated strain | I | 6000 | | 1000 |
| *Staphylococcus hominis* | YS3 | Isolated strain | J | 6000 | | 1000 |
| *Salmonella* | LHL37207 | Isolated strain | K | 1200 | | 200 |
|  | LHL40665 | Isolated strain |  | 1200 | | 200 |
|  | LHL1 | Isolated strain |  | 1200 | | 200 |
|  | LHL2 | Isolated strain |  | 1200 | | 200 |
|  | LHL3 | Isolated strain |  | 1200 | | 200 |
| *Kocuria rhizophila* | CMCC28001 | Reference strain | L | 6000 | | 1000 |
| *Staphylococcus lentus* | CICC21602 | Reference strain | M | 6000 | | 1000 |
| *Escherichia coli* | KW03004 | Isolated strain | N | 300 | | 50 |
|  | KW03005 | Isolated strain |  | 300 | | 50 |
|  | KW03006 | Isolated strain |  | 300 | | 50 |
|  | KW03007 | Isolated strain |  | 300 | | 50 |
|  | KW03008 | Isolated strain |  | 300 | | 50 |
|  | KW03010 | Isolated strain |  | 300 | | 50 |
|  | KW03011 | Isolated strain |  | 300 | | 50 |
|  | KW03012 | Isolated strain |  | 300 | | 50 |
|  | KW03013 | Isolated strain |  | 300 | | 50 |
|  | KW03021 | Isolated strain |  | 300 | | 50 |
|  | KW03025 | Isolated strain |  | 300 | | 50 |
|  | KW03038 | Isolated strain |  | 300 | | 50 |
|  | KW03076 | Isolated strain |  | 300 | | 50 |
|  | CMCC44102 | Reference strain |  | 300 | | 50 |
|  | CMCC44113 | Reference strain |  | 300 | | 50 |
|  | CMCC44829 | Reference strain |  | 300 | | 50 |
|  | LHL40179 | Isolated strain |  | 300 | | 50 |
|  | LHL40245 | Isolated strain |  | 300 | | 50 |
|  | LHL40505 | Isolated strain |  | 300 | | 50 |
|  | LHL40540 | Isolated strain |  | 300 | | 50 |
