# Supplementary material for: Helix Matrix Transformation Combined With Convolutional Neural Network Algorithm for Matrix-Assisted Laser Desorption Ionization-Time of Flight Mass Spectrometry-Based Bacterial Identification
Source: Front Microbiol. 2020 Nov 12;11:565434. doi: 10.3389/fmicb.2020.565434 (PMC7693542; doi:10.3389/fmicb.2020.565434)
Supplement: Supplementary file 3 [file Table_2.docx]

Table S2. Structure and parameters of convolutional neural network

| Layers | Parameters | Activation |
| --- | --- | --- |
| InputLayer | Shape=[25,25,1] |  |
| Conv2D | Filter=4/kernel=3 | ReLU |
| BatchNormalization |  |  |
| Conv2D | Filter=8/kernel=3 | ReLU |
| BatchNormalization |  |  |
| MaxPooling2D |  |  |
| Conv2D | Filter=16/kernel=3 | ReLU |
| Flatten |  |  |
| Dense | 64 | ReLU |
| Dense | 14 | Softmax |
